# Metformin Targets Foxo1 to Control Glucose Homeostasis

**DOI:** 10.3390/biom11060873

**Published:** 2021-06-11

**Authors:** Xiaoqin Guo, Xiaopeng Li, Wanbao Yang, Wang Liao, James Zheng Shen, Weiqi Ai, Quan Pan, Yuxiang Sun, Kebin Zhang, Rui Zhang, Yuyang Qiu, Qian Dai, Hongting Zheng, Shaodong Guo

**Affiliations:** 1 Xinqiao Hospital, Army Medical University, Chongqing 400037, China; Janeguo4@163.com (X.G.); zhangkebin12@163.com (K.Z.); zhangrui_nesta@163.com (R.Z.); qyy19881110@163.com (Y.Q.); daiqianzy@163.com (Q.D.); 2 Department of Nutrition, College of Agriculture and Life Science, Texas A&M University, College Station, TX 77843, USA; xiaopengli@zju.edu.cn (X.L.); wanbao09@tamu.edu (W.Y.); wangliao@seu.edu.cn (W.L.); jamesshen@tamu.edu (J.Z.S.); awq123007@tamu.edu (W.A.); beanshe@tamu.edu (Q.P.); yuxiangs@tamu.edu (Y.S.)

**Keywords:** metformin, salicylate, Foxo1-S273 phosphorylation, type 2 diabetes, glucose homeostasis

## Abstract

Metformin is the first-line pharmacotherapy for type 2 diabetes mellitus (T2D). Metformin exerts its glucose-lowering effect primarily through decreasing hepatic glucose production (HGP). However, the precise molecular mechanisms of metformin remain unclear due to supra-pharmacological concentration of metformin used in the study. Here, we investigated the role of Foxo1 in metformin action in control of glucose homeostasis and its mechanism via the transcription factor Foxo1 in mice, as well as the clinical relevance with co-treatment of aspirin. We showed that metformin inhibits HGP and blood glucose in a Foxo1-dependent manner. Furthermore, we identified that metformin suppresses glucagon-induced HGP through inhibiting the PKA→Foxo1 signaling pathway. In both cells and mice, Foxo1-S273D or A mutation abolished the suppressive effect of metformin on glucagon or fasting-induced HGP. We further showed that metformin attenuates PKA activity, decreases Foxo1-S273 phosphorylation, and improves glucose homeostasis in diet-induced obese mice. We also provided evidence that salicylate suppresses HGP and blood glucose through the PKA→Foxo1 signaling pathway, but it has no further additive improvement with metformin in control of glucose homeostasis. Our study demonstrates that metformin inhibits HGP through PKA-regulated transcription factor Foxo1 and its S273 phosphorylation.

## 1. Introduction

The global prevalence of diabetes mellitus has been rising rapidly, and the number of patients with diabetes mellitus has quadrupled during the past 3 decades [1]. Metformin is currently the first-line drug for the treatment of type 2 diabetes (T2D), primarily owing to its function in reducing HGP [2,3]. However, the underlying mechanisms of metformin action remain incompletely understood.

Metformin belongs to the biguanide class originally found from the plant *Galega officinalis* [4]. Metformin alleviates hyperglycemia in T2D primarily through suppression of HGP [5] via several mechanisms [6,7,8]. Metformin, at low concentration, activates AMPK, which triggers CBP-CREB-TORC2 complex dissociation and decreases glucagon-induced cAMP accumulation through cyclic nucleotide phosphodiesterase-4B (PDE4B) [6,9]. A low-dose metformin treatment also reduces endogenous HGP through inhibiting mitochondrial glycerophosphate dehydrogenase [10]. Moreover, a high concentration of metformin contributes to a significant decrease in ATP and increase in AMP levels [11,12]. The increased AMP/ATP ratio enhances AMPK activity and the elevated AMP also inhibits adenylate cyclase, thus reducing cAMP levels and suppressing PKA activity, which blocks glucagon-induced HGP [13]. Depletion of ATP by metformin also contributes to the suppression of hepatic gluconeogenesis [11]. AMP also inhibits the activity of fructose −1,6-bisphophatase-1 (FBP1), a rate-controlling enzyme in gluconeogenesis [14]. Therefore, metformin with different concentrations targets different molecules, whereby it suppresses HGP.

Aspirin, a widely used drug for the treatment of a number of diseases, including fever, pain, rheumatic fever, and inflammatory conditions. Salicylate, an active metabolite of aspirin, is commonly claimed to associate with suppression of protein kinase IκB kinase β (IKKβ) in the NF-κB pathway, reducing inflammation in patients with T2D [15,16,17,18]. Salicylate activates AMPK and inhibits dephosphorylation of AMPKα-T172 and improves metformin resistance [17,19], suggesting that salicylate might mitigate the glucagon-controlled glucose metabolism and glucose homeostasis.

Foxo1, a member of the O-class forkhead/winged helix transcriptional factor, is a key regulator in control of gluconeogenesis [20,21,22]. Recently, we established that glucagon-stimulated PKA promotes Foxo1 phosphorylation at S273, promoting Foxo1 nucleus translocation and enhancing gluconeogenesis [23]. However, whether metformin-regulated HGP relies on Foxo1 and how metformin regulates Foxo1 remains unknown. In this study, we detected the effect of metformin on glucagon-induced HGP and blood glucose with different doses. We found that metformin inhibits PKA activity, decreases Foxo1-S273 phosphorylation, and attenuates Foxo1 activity, contributing to its suppression of HGP and blood glucose. In addition, salicylate also regulates glucose homeostasis through the PKA→Foxo1 signaling pathway and has no further additive effect on metformin action on blood glucose regulation.

## 2. Materials and Methods

### 2.1. Patient Recruitment and Clinical Investigation

Clinical observations and intervention were initiated from April 2017 to November 2017 in the Department of Endocrinology, Xinqiao Hospital, Chongqing, China. All subjects submitted their informed consent for participation before the study initiated. The study was conducted in accordance with the Declaration of Helsinki, and the protocol was approved by the Ethics Committee of Institute Review Board (IRB) of Xinqiao Hosptial, Chongqing. Both male and female patients with type 2 diabetes were recruited with average age of 53 years old (*n* = 76). Most of them were newly diagnosed with T2D. They were randomly divided into two groups and treated with metformin (group M) and metformin + aspirin (group M + A) in the therapeutic doses for 4 weeks. The metformin-treated group orally took 0.85 g of metformin twice daily, while the metformin + aspirin group orally took 0.85 g of metformin twice daily and a 100 mg aspirin tablet daily. Patients who suffered from other complications, such as diarrhea, and those who could not be contacted after 4 weeks of treatment were excluded. The blood samples of patients were collected before and after 4 weeks of treatment, and blood profiles were determined, including HbA1c, blood glucose, insulin, glucagon, high-density lipoprotein (HDL), low-density lipoprotein (LDL), triglyceride, and free fatty acid. (Metformin group: *n* = 8. Metformin + Aspirin group: *n* = 12.) All protocols of clinical study were approved by the Ethics Committee of the Second Affiliated Hospital of Army Medical University, Chongqing, China.

### 2.2. Animal Experiments

All animal protocols were approved by the Institutional Animal Care and Use Committee of Texas A&M University. Liver-specific Foxo1 knock out (FKO), *Foxo1*^S273A^, and *Foxo1*^273D^ mice were generated as previously described [23,24]. All experiments were conducted in male mice. For the metformin/salicylate tolerance test, the mice were fasted for 16 h, then were injected intraperitoneally (i.p.) with 100, 150, and 200 mg/kg metformin (ENZO Life Sciences, Farmingdale, NY, USA), 200 mg/kg salicylate (Sigma, St. Louis, MO, USA), or saline. The blood glucose was measured at indicated time points. For chronic study, mice were daily given metformin (50 mg/kg), salicylate (125 mg/kg), or the combination (50 mg/kg metformin + 125 mg/kg salicylate) by oral administration (9:00–10:30 a.m.) for 4 weeks. The fasting blood glucose was measured every week. For the pyruvate tolerance test, the control and FKO mice were fasted for 16 h at the fifth week and i.p. injected with 2 g/kg body weight pyruvate. Blood glucose was monitored. Eight to twelve-week old control and FKO mice were fed a standard chow diet (54% calories from carbohydrate, 14% from fat, and 32% from protein) (Envigo Teklad Diet, 3.0 kcal/g) ad libitum. High-fat diet (HFD) mice were purchased from Jackson Lab (Bar Harbor, ME, USA). Mice were fed with HFD (60% research diet) for 6 weeks, then administered drinking water containing 0.4 mg/mL metformin (equivalent to 50 mg/kg body weight metformin), 1.25 mg/mL salicylate (equivalent to 125 mg/kg body weight salicylate), or 0.4 mg/mL metformin + 1.25 mg/mL salicylate for 7 weeks [25,26]. After 16 h of fasting, pyruvate tolerance tests (2 g/kg body weight; i.p. injection) and glucose tolerance tests (2 g/kg body weight; i.p. injection) were performed to evaluate the glucose homeostasis in mice.

### 2.3. Hepatic Glucose Production Assay

Primary hepatocytes were isolated from WT or FKO and seeded to the collagen-coated 6-well plate with 300,000 cells per well [24,27]. Primary hepatocytes were incubated in low glucose DMEM (2% FBS), 37 °C for 3 h for cell attachment. After attachment, the medium was replaced by HGP buffer (119 mM NaCl, 5 mM KCl, 2.6 mM KH_2_PO_4_, 2.6 mM MgSO_4_, 2 mM CaCl_2_, 24.6 mM NaHCO_3_, 10 mM HEPES, 0.5%BSA, 10 mM Lactate, 5 mM Pyruvate). Metformin and salicylate were pretreated in corresponding groups for 30 min, followed by 100 nM glucagon treatment for 3 h. Glucose content in medium was determined by Amplex™ Red Glucose/Glucose Oxidase Assay Kit (Thermo Fisher, Waltham, MA, USA) according to manufactory protocol and normalized by protein.

### 2.4. Western Blotting

The proteins were extracted from primary hepatocyte or liver, and an equal amount of protein was loaded in SDS-PAGE for Western blot. Antibodies for Foxo1, GAPDH, and PKA substrates phosphorylation were purchased from Cell Signaling Technology (Danvers, MA, USA). G6PC antibody was purchased from Abcam (Cambridge, MA, USA). Phosphorylated Foxo1 at S273 antibody was generated as previously reported [23].

### 2.5. Protein Kinase a Activity Assay

Mouse primary hepatocytes were isolated as mentioned, and the medium was changed to HGP buffer after 3 h attachment. Metformin and salicylate were pretreated for 30 min before glucagon (100 nM) was added. The samples were collected after 1 h treatment. The PKA activity was measured using the PKA kit (Thermo Scientific, Waltham, MA, USA) according to the manufactory protocol and normalized by protein.

### 2.6. Insulin and Glucagon Measurement

Serum insulin and glucagon levels were measured using the Mouse Insulin ELISA kit (Mercodia, Sweden) and Mouse Glucagon ELISA kit (Crystal Chem, Elk Grove Village, IL, USA), according to the manual instructions.

### 2.7. Real-Time Quantitative PCR

Liver RNA was extracted with Trizol reagent (Invitrogen, Carlsbad, CA, USA), and cDNA was synthesized using iScript cDNA Synthesis kit (Bio-Rad Laboratories Inc., Hercules, CA, USA). Gene expression was measured using the SYBR Green Supermix according to the manufacturer’s protocol in real-time PCR system (Bio-Rad Laboratories Inc., Hercules, CA, USA). Real-time PCR primers are listed as follows: iNOS: F: TCCTGGAGGAAGTGGGCCGAAG, R: CCTCCACGGGCCCGGTACTC; TNFα: F: GAGAAAGTCAACCTCCTCTCTG, R: GAAGACTCCTCCCAGGTATATG; MCP1: F: CAGGTGTCCCAAAGAAGCTGTAG, R: GGGTCAGCACAGACCTCTCTCT.

### 2.8. Statistical Analysis

All results are presented as mean ± SEM. *p* values were calculated using Student’s *t*-test for the comparison of differences between two groups. Significance among multiple groups was tested using one-way or two-way ANOVA. *p* < 0.05 was considered statistically significant.

## 3. Results

### 3.1. Metformin Suppresses Glucagon-Induced HGP through PKA-Foxo1 Pathway in Hepatocytes

**Figure 1:**
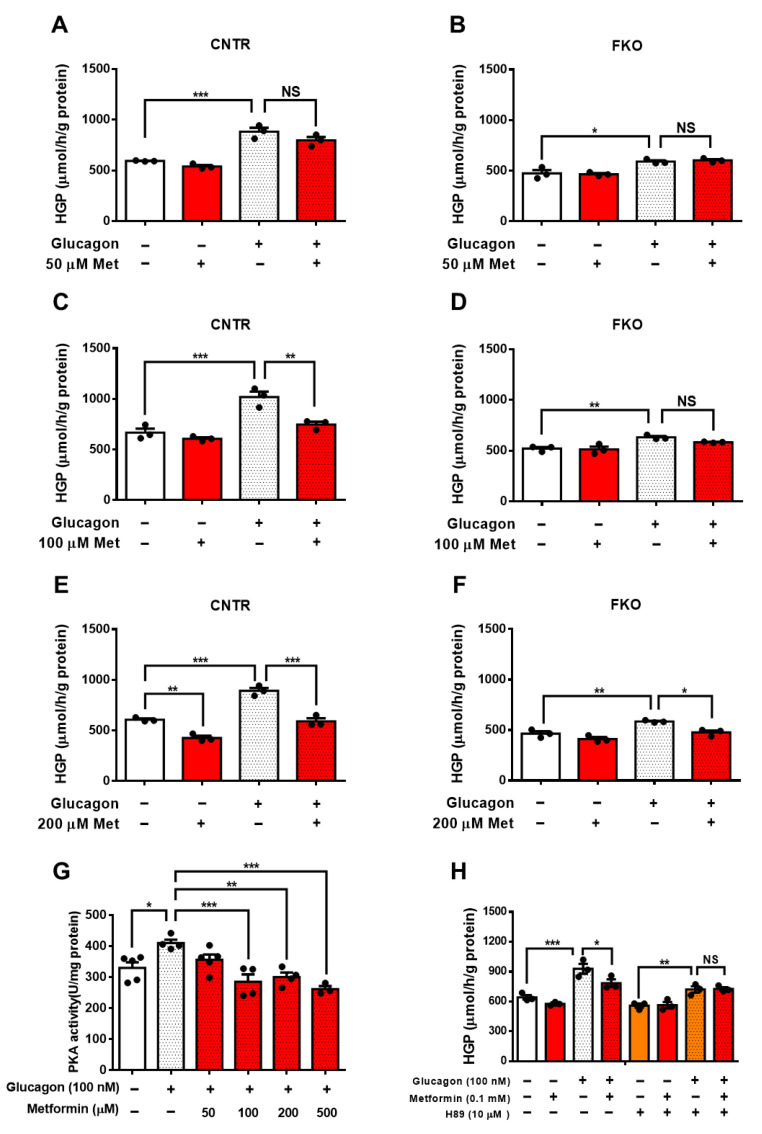
Metformin suppresses glucagon-induced HGP through the PKA–Foxo1 signaling pathway in primary hepatocytes. (**A**,**B**) Primary hepatocytes isolated from control (**A**) and FKO (**B**) mice were pretreated with 50 μM of metformin for 30 min and treated with 100 nM of glucagon for 3 h. Glucose content was measured, *n* = 3 independent experiments/group. (**C**,**D**) Primary hepatocytes isolated from control (**C**) and FKO (**D**) mice were pretreated with 100 μM of metformin for 30 min and treated with 100 nM of glucagon for 3 h. Glucose content was measured, *n* = 3 independent experiments/group. (**E**,**F**) Primary hepatocytes isolated from control (**E**) and FKO (**F**) mice were pretreated with 200 μM of metformin for 30 min and treated with 100 nM of glucagon for 3 h. Glucose content was measured, *n* = 3 independent experiments/group. (**G**) Control hepatocytes were pretreated with 50, 100, 200, and 500 μM of metformin for 30 min and treated with 100 nM of glucagon for 1 h. PKA activity was analyzed, *n* = 3–5 independent experiments/group. (**H**) Control hepatocytes were pretreated with H89 and metformin, followed by 100 nM glucagon treatment for 3 h. Glucose content was measured, *n* = 3 independent experiments/group. All are are presented as mean ± SEM. * *p* < 0.05, ** *p* < 0.01, *** *p* < 0.001, NS: no significance, CNTR: control.

To detect whether metformin-mediated HGP suppression requires Foxo1, we treated control and Foxo1 knockout (FKO) primary hepatocytes with different doses of metformin. In control hepatocytes, glucagon significantly increased HGP and 50 μM metformin treatment led to an insignificant reduction in glucagon-induced HGP (Figure 1A). In FKO hepatocytes, glucagon also significantly elevated HGP, whereas 50 μM of metformin had no effect on glucagon-induced HGP (Figure 1B). We then treated hepatocytes with 100 μM of metformin and found that 100 μM metformin treatment significantly abolished glucagon-induced HGP by 27% in control hepatocytes (Figure 1C); however, 100 μM of metformin barely affected glucagon-induced HGP in FKO hepatocytes (Figure 1D). Treatment with 200 μM of metformin significantly reduced glucagon-induced HGP by 34% in control hepatocytes (Figure 1E). In FKO hepatocytes, 200 μM of metformin significantly resulted in a 19% reduction in glucagon-induced HGP (Figure 1F). PKA, a key player in glucagon signaling in control of glucose metabolism, promotes Foxo1 activity via S273 phosphorylation [23]. We then investigated whether PKA is involved in the metformin-induced HGP suppression. Glucagon significantly increased PKA activity by 24% in control hepatocytes. Treatment with 50 μM of metformin led to an insignificant inhibition on PKA activity upon glucagon challenge; however, 100, 200, and 500 μM metformin treatment significantly suppressed glucagon-induced HGP by 30%, 30%, and 36%, respectively (Figure 1G). Consistently, 100 μM of metformin significantly attenuated glucagon-induced HGP in control hepatocytes, whereas inhibition of PKA by H89 blocked the suppressive effect of metformin on HGP upon glucagon treatment (Figure 1H). Collectively, these results indicate that metformin suppresses glucagon-induced HGP partially through PKA-Foxo1 pathway in hepatocytes.

### 3.2. Metformin Inhibits Blood Glucose and HGP through Foxo1 in Mice

**Figure 2:**
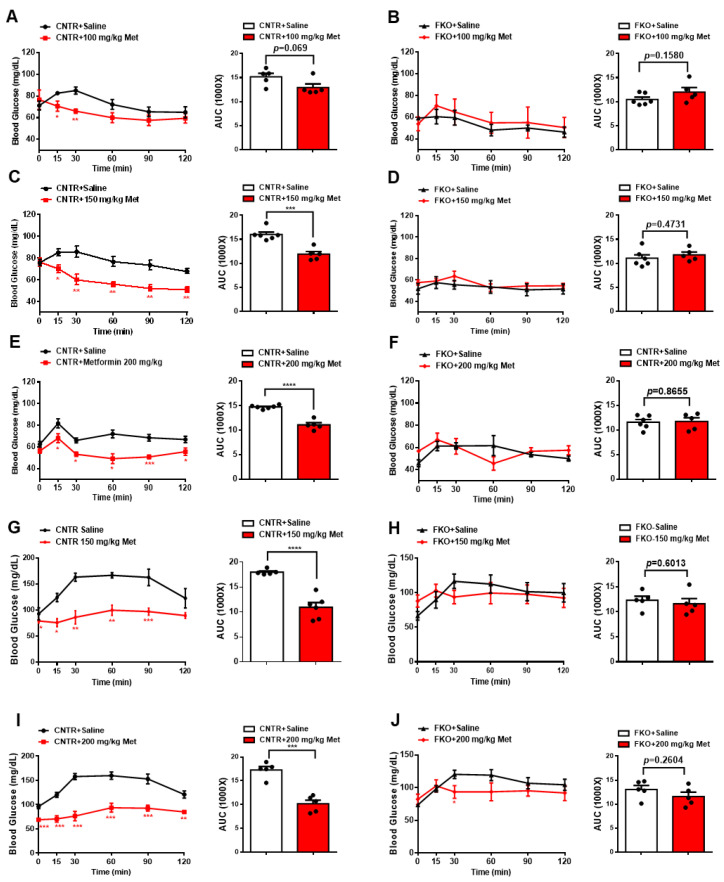
Metformin suppresses blood glucose in mice through hepatic Foxo1 gene. (**A**,**B**) 100 mg/kg body weight metformin (Met) was acutely i.p. injected into control (**A**)and FKO (**B**) mice. Blood glucose was monitored at indicated time points, *n* = 5–6 mice/group. (**C**,**D**) 150 mg/kg body weight metformin (Met) was acutely i.p. injected into control (**C**) and FKO (**D**) mice. Blood glucose was monitored at indicated time points, *n* = 5–6 mice/group. (**E**,**F**) 200 mg/kg body weight metformin (Met) was acutely i.p. injected into control (**E**) and FKO (**F**) mice. Blood glucose was monitored at indicated time points, *n* = 5–6 mice/group. (**G**,**H**) 150 mg/kg body weight metformin (Met) was acutely i.p. injected into control (**G**) and FKO (**H**) mice after 16 h fasting. After 45 min, pyruvate tolerance test was performed, *n* = 5–6 mice/group. (**I**,**J**) Control (**I**) and FKO (**J**) mice were fasted for 16 h and then i.p. injected with 200 mg/kg body weight metformin. After 45 min, pyruvate tolerance test was performed, *n* = 5 mice/group. All data are presented as mean ± SEM. *** *p* < 0.001, **** *p* <0.0001. CNTR: control.

We then examined whether metformin lowers blood glucose and HGP through hepatic Foxo1 in mice. Here, we used control (floxed Foxo1) and hepatic Foxo1 gene knockout (FKO) mice [22]. Intraperitoneal (i.p.) injections of 100, 150, and 200 mg/kg body weight metformin decreased fasting blood glucose by 15%, 26%, and 25%, respectively, in control mice (Figure 2A,C,E). However, hepatic Foxo1 deficiency diminished the suppressive effect of metformin on fasting blood glucose in FKO mice (Figure 2B,D,F). Consistently, i.p. injection of 150 and 200 mg/kg body weight metformin significantly decreased glucose output upon pyruvate challenge in control mice by 39% and 41%, respectively (Figure 2G,I). However, both 150 and 200 mg/kg body weight metformin administration had no effect on pyruvate-induced glucose output in FKO mice (Figure 2H,J). Thus, these results suggest that hepatic Foxo1 is required for the suppressive effect of metformin on blood glucose and glucose output in mice.

### 3.3. Foxo1-S273 Mutations Attenuate the Suppressive Effect of Metformin on HGP in Both Hepatocytes and Mice

**Figure 3:**
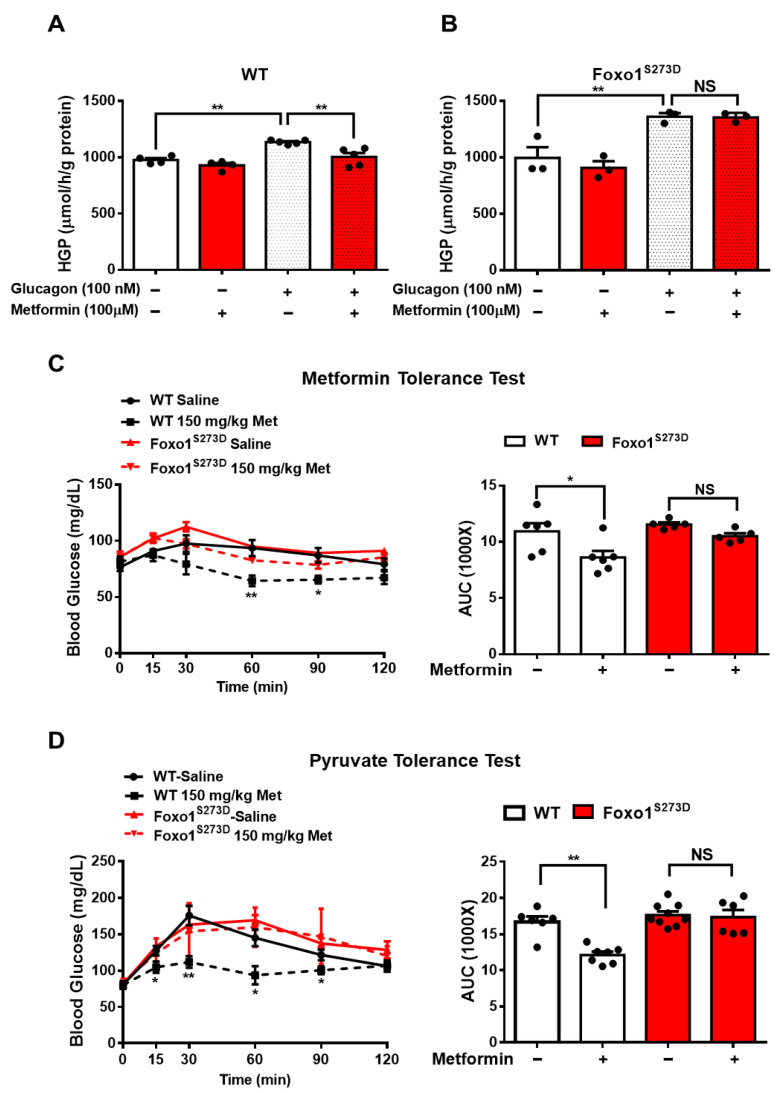
Foxo1-S273D mutation blocks metformin-induced HGP and blood glucose suppression in mice. (**A**,**B**) Primary hepatocytes isolated from WT (**A**) and Foxo1^S273D^ (**B**) mice were pretreated with 100 μM of metformin for 30 min and then treated with 100 nM of glucagon for 3 h. Glucose content was measured, *n* = 3–4 independent experiments/group. (**C**) 150 mg/kg body weight metformin (Met) was acutely i.p. injected into 16 h fasting WT and Foxo1^S273D^ mice. Blood glucose was monitored at indicated time points, *n* = 5–6 mice/group. (**D**) WT and Foxo1^S273D^ mice were fasted for 16 h and then i.p. injected with 150 mg/kg body weight metformin. After 45 min, pyruvate tolerance test was performed, *n* = 6–9 mice/group. All data are presented as mean ± SEM. * *p* < 0.05, ** *p* < 0.01, NS: no significance.

Given that PKA increases Foxo1 activity through Foxo1-S273 phosphorylation, as we previously demonstrated [23], we further evaluated whether Foxo1-S273 phosphorylation plays a key role in metformin-induced HGP suppression. Here, we used Foxo1-S273D or A mutation mouse, where serine (S) was replaced by aspartic acid (D) or alanine (A) to mimic constitutive phosphorylation or dephosphorylation, respectively [23]. Consistently, in wild type (WT) hepatocytes, 100 μM metformin treatment significantly attenuated glucagon-induced HGP by 16% (Figure 3A); however, in Foxo1^S273D^ hepatocytes, the suppressive effect of metformin was abolished (Figure 3B). The metformin tolerance test in mice indicated that 150 mg/kg metformin i.p. injection significantly decreased fasting blood glucose by 21% in WT mice, whereas Foxo1-S273D mutation blocked the effect of metformin on reducing blood glucose in mice (Figure 3C). Further analysis of pyruvate-induced glucose production showed that 150 mg/kg metformin injection significantly reduced blood glucose by 28% during pyruvate tolerance test in WT mice; such an effect was blocked in Foxo1-S273D mutant mice (Figure 3D).

**Figure 4:**
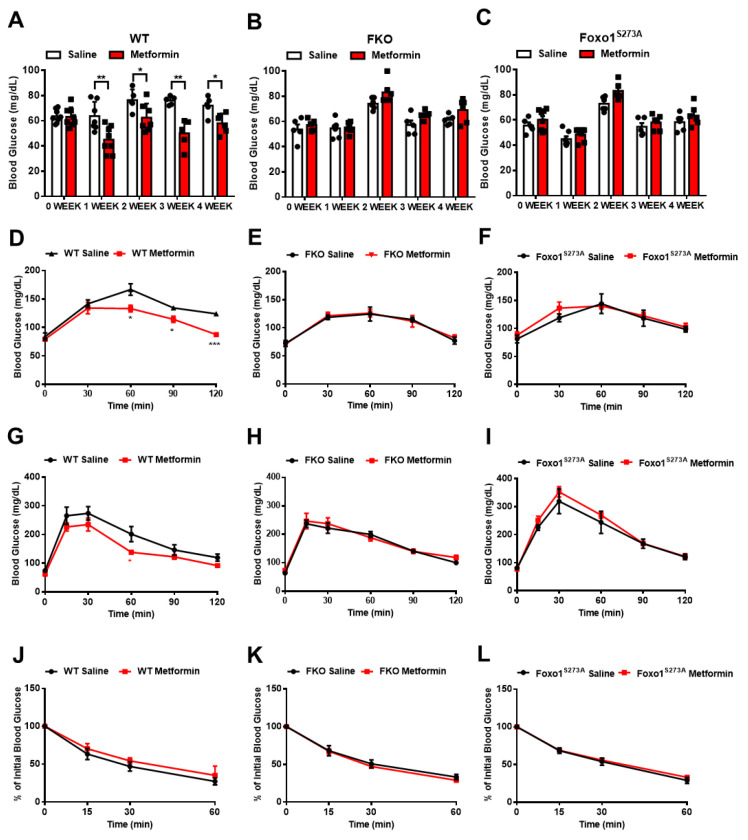
Chronic metformin treatment reduces blood glucose through interference of Foxo1-S273 phosphorylation. (**A**–**C**) Blood glucose in WT (**A**), FKO (**B**), and Foxo1^S273A^ (**C**) mice chronically treated with 50 mg/kg body weight metformin after 16 h fasting, *n* = 5–7 mice/group. (**D**–**F**) Pyruvate tolerance test in WT (**D**), FKO (**E**), and Foxo1^S273A^ (**F**) mice chronically treated with 50 mg/kg body weight metformin, *n* = 5–7 mice/group. (**G**–**I**) Glucose tolerance test in WT (**G**), FKO (**H**), and Foxo1^S273A^ (**I**) mice chronically treated with 50 mg/kg body weight metformin, *n* = 5–7 mice/group. (**J**–**L**) Insulin tolerance test in WT (**J**), FKO (**K**), and Foxo1^S273A^ (**L**) mice chronically treated with 50 mg/kg body weight metformin, *n* = 5–7 mice/group. All data are presented as mean ± SEM. * *p* < 0.05, ** *p* < 0.01, *** *p* < 0.001.

We then treated mice with 50 mg/kg body weight metformin via daily oral injection for 4 weeks and evaluated glucose homeostasis after a chronic treatment. In control mice, the chronic treatment of metformin significantly reduced fasting blood glucose (Figure 4A) and decreased pyruvate-induced glucose production (Figure 4D). Importantly, in either FKO or Foxo1^S273A^ mice, the chronic treatment of metformin had no effect on reducing fasting blood glucose (Figure 4B,C) and glucose output upon pyruvate challenge (Figure 4E,F). We further detected the effect of metformin on glucose tolerance in mice. In order to reduce variability in baseline blood glucose and insulin levels, we performed overnight (16 h) fasting to mobilize the glucose reservoirs and deplete liver glycogen stores. The control mice with chronic metformin treatment showed a significant improvement in glucose tolerance (Figure 4G); however, both FKO and Foxo1^S273A^ mice had no significant changes in glucose tolerance in response to chronic metformin treatment (Figure 4H,I). Insulin sensitivity was not affected by chronic treatment of metformin in control, FKO, and Foxo1^S273A^ mice (Figure 4J–L). These results indicate that metformin decreases blood glucose and HGP largely through Foxo1 and Foxo1-S273 phosphorylation.

### 3.4. Salicylate Inhibits Glucagon-Induced HGP via Foxo1 and Has No Improvement on Metformin-Mediated HGP Suppression

**Figure 5:**
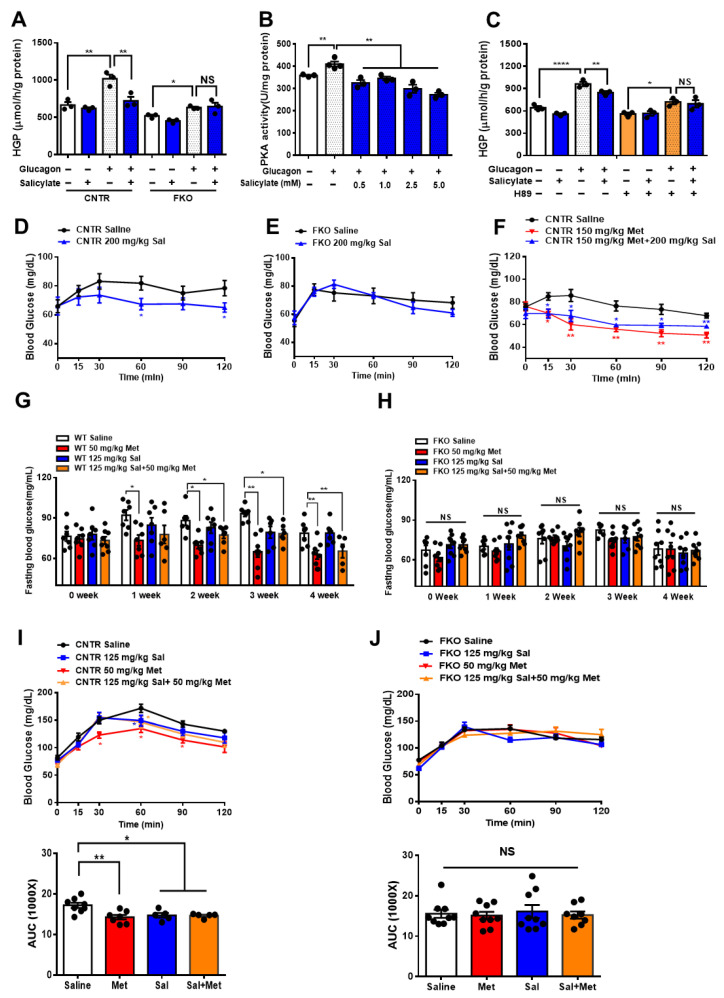
Salicylate suppresses glucagon-induced HGP through the PKA–Foxo1 signaling pathway in hepatocytes and has no improvement on metformin action on glucose homeostasis. (**A**) Primary hepatocytes isolated from control and FKO mice were pretreated with 0.5 mM of salicylate for 30 min and treated with 100 nM of glucagon for 3 h. Glucose content was measured, *n* = 3 independent experiments/group. (**B**) Control primary hepatocytes were pretreated with 0.5, 1, 2.5, and 5 mM of salicylate for 30 min, followed by 100 nM treatment for 1 h. PKA activity was measured, *n* = 3–4. (**C**) Control hepatocytes were pretreated with H89 and 0.5 mM of salicylate, followed by 100 nM glucagon treatment for 3 h. Glucose content was measured, *n* = 3 independent experiments/group. (**D**,**E**) Control (**D**) and FKO (**E**) mice were i.p. injected with 200 mg/kg body weight salicylate (Sal) after 16 h fasting. Blood glucose was monitored at indicated time points, *n* = 5–9 mice/group. (**F**) Control mice were i.p. injected with 150 mg/kg body weight metformin (Met) or 150 mg/kg body weight metformin (Met) + 200 mg/kg body weight salicylate (Sal) after 16 h fasting. Blood glucose was measured at indicated time points, *n* = 5–6 mice/group. (**G**,**H**) Control (**G**) and FKO (**H**) mice were administered with 50 mg/kg body weight metformin (Met), 125 mg/kg body weight salicylate (Sal), or 50 mg/kg body weight Met + 125 mg/kg body weight Sal via oral injection. Blood glucose was monitored after 16 h fasting, *n* = 5–8 mice/group. (**I**,**J**) Pyruvate tolerance test in control (**I**) and FKO (**J**) mice administered with 50 mg/kg body weight metformin (Met), 125 mg/kg body weight salicylate (Sal), or 50 mg/kg body weight Met + 125 mg/kg body weight Sal via oral injection, *n* = 5–8 mice/group. All data are presented as mean ± SEM. * *p* < 0.05, ** *p* < 0.01, **** *p* <0.0001, NS: no significance, CNTR: control.

It is known that salicylate treatment improves glucose homeostasis and insulin sensitivity [28,29,30]. We next examined whether salicylate treatment enhances the beneficial effect of metformin on the regulation of glucose metabolism. We firstly treated the control and FKO hepatocytes with salicylate and found that salicylate significantly abolished glucagon-induced HGP; however, hepatic Foxo1 deficiency blocked the suppressive effect of salicylate on HGP upon glucagon treatment (Figure 5A). Indeed, salicylate treatment markedly decreased glucagon-induced PKA activity (Figure 5B) and inhibition of PKA by H89 diminished the salicylate-mediated HGP suppression in control hepatocytes treated with glucagon (Figure 5C). Consistently, acute salicylate i.p. injection significantly decreased fasting blood glucose in control mice (Figure 5D); such an effect was blocked in FKO mice (Figure 5E). However, salicylate administration did not enhance the suppressive effect of metformin on fasting blood glucose in control mice (Figure 5F). We then treated mice with 50 mg/kg body weight metformin and 125 mg/kg body weight salicylate through daily oral injection for 4 weeks. Consistently, chronic treatment of metformin significantly decreased fasting blood glucose in control mice, which was abolished in FKO mice. Salicylate treatment had little effect on fasting blood glucose and did not further enhance metformin-mediated reduction in fasting blood glucose in control mice. Hepatic Foxo1 deficiency blocked the suppressive effect of both metformin and salicylate on fasting blood glucose (Figure 5G,H). Chronic treatment of metformin and salicylate significantly deceased pyruvate-induced HGP by 17% and 14%, respectively, in control mice; however, salicylate treatment did not further enhance the metformin-mediated HGP suppression (Figure 5I). The inhibitory effects of metformin and salicylate on HGP during pyruvate tolerance test were diminished in FKO mice (Figure 5J). Thus, these results indicated that salicylate suppresses HGP through Foxo1 without further improvement on metformin-mediated HGP suppression.

### 3.5. Metformin and Salicylate Improve HFD-induced Glucose Dysregulation through PKA-Foxo1 Pathway

**Figure 6:**
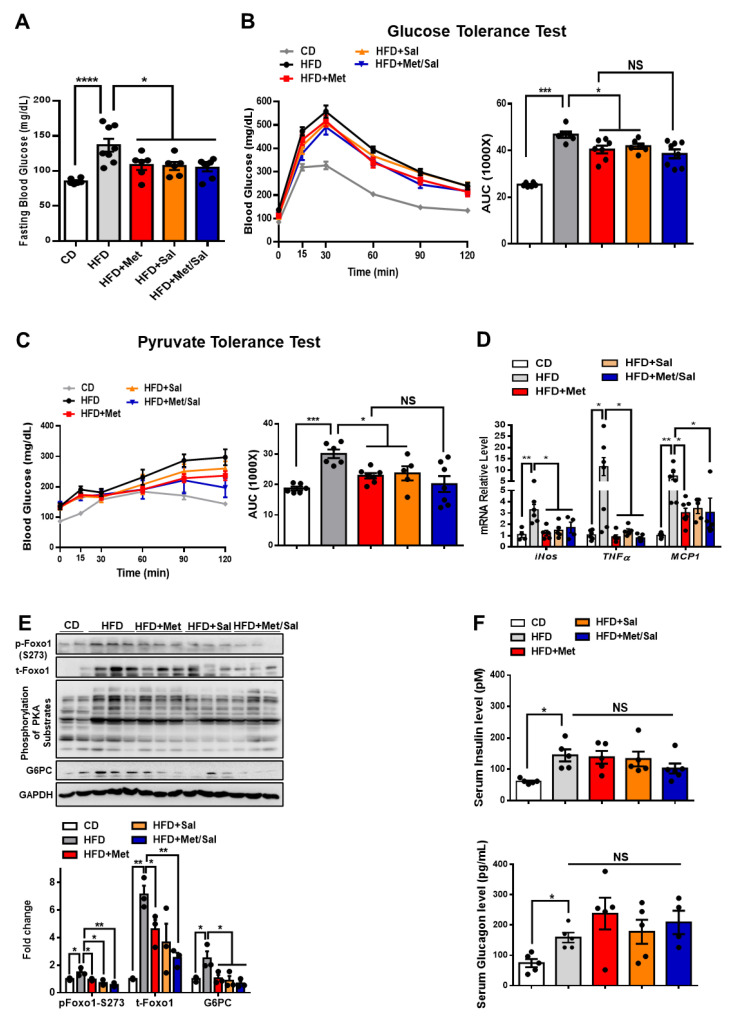
Metformin and salicylate improve glucose homeostasis in HFD-fed mice. (**A**) Fasting blood glucose level in HFD mice treated with metformin and salicylate for 7 weeks after 16 h fasting, *n* = 6–8 mice/group. (**B**) Glucose tolerance test in HFD mice treated with metformin and salicylate for 7 weeks, *n* = 6–8 mice/group. (**C**) Pyruvate tolerance test in HFD mice treated with metformin and salicylate for 7 weeks, *n* = 6–8 mice/group. (**D**) The mRNA expression levels of inflammatory factors in livers of 16 h overnight fasted HFD mice treated with metformin and salicylate for 7 weeks, *n* = 4–6 mice/group. (**E**) The protein levels of gluconeogenic genes in livers of 16 h overnight fasted HFD mice treated with metformin and salicylate for 7 weeks, *n* = 3 mice/group. (**F**) The insulin and glucagon levels in serum from HFD-fed mice treated with metformin and salicylate for 7 weeks, *n* = 4–5 mice/group. All data are presented as mean ± SEM. * *p* < 0.05, ** *p* < 0.01, *** *p* < 0.001, **** *p* < 0.0001. CD: chow diet.

We next evaluated the roles of metformin and salicylate in glucose homeostasis in diet-induced obese mice. The WT mice were fed with HFD for 6 weeks and then treated with metformin and salicylate for 7 weeks under HFD feeding. Both metformin and salicylate administrations significantly decreased fasting blood glucose levels by 20% and 21%, respectively, in the HFD-fed mice. However, salicylate treatment did not further enhance the suppressive effect of metformin on fasting blood glucose (Figure 6A). Consistently, the HFD feeding significantly impaired glucose tolerance and pyruvate tolerance, as compared with normal chow diet-fed control mice, and the effect of HFD was partially rescued by metformin or salicylate treatment. However, co-treatment of metformin and salicylate only resulted in an insignificant improvement in glucose tolerance and pyruvate-induced glucose output, as compared to metformin treatment group alone (Figure 6B,C). In addition to the anti-hyperglycemic effect, metformin showed a comparable effect in reducing the mRNA levels of pro-inflammatory markers in the liver of HFD-fed mice as compared to the salicylate treatment, but there was no enhanced effect of the combined treatment (Figure 6D). We further analyzed the protein expression in livers and found that HFD feeding activated the PKA→Foxo1-S273 phosphorylation; this was evident by increased levels of pan-PKA substrates’ phosphorylation, pFoxo1-S273, and protein abundance of total Foxo1 and G6PC (Figure 6E). Of note, metformin or/and salicylate alleviated the activation of the hepatic PKA→Foxo1 signaling pathway in the HFD-fed mice with a modest additive effect (Figure 6E). In addition, neither metformin nor salicylate treatment significantly altered serum insulin and glucagon levels (Figure 6F). Collectively, the above results suggest that metformin and salicylate improved glucose homeostasis through the PKA→Foxo1 signaling pathway and salicylate treatment does not significantly enhance the beneficial effect of metformin on glucose regulation in HFD-fed mice.

### 3.6. The Effect of Metformin and Aspirin Combined Therapy on Blood Profiles in T2D Patients

**Figure 7:**
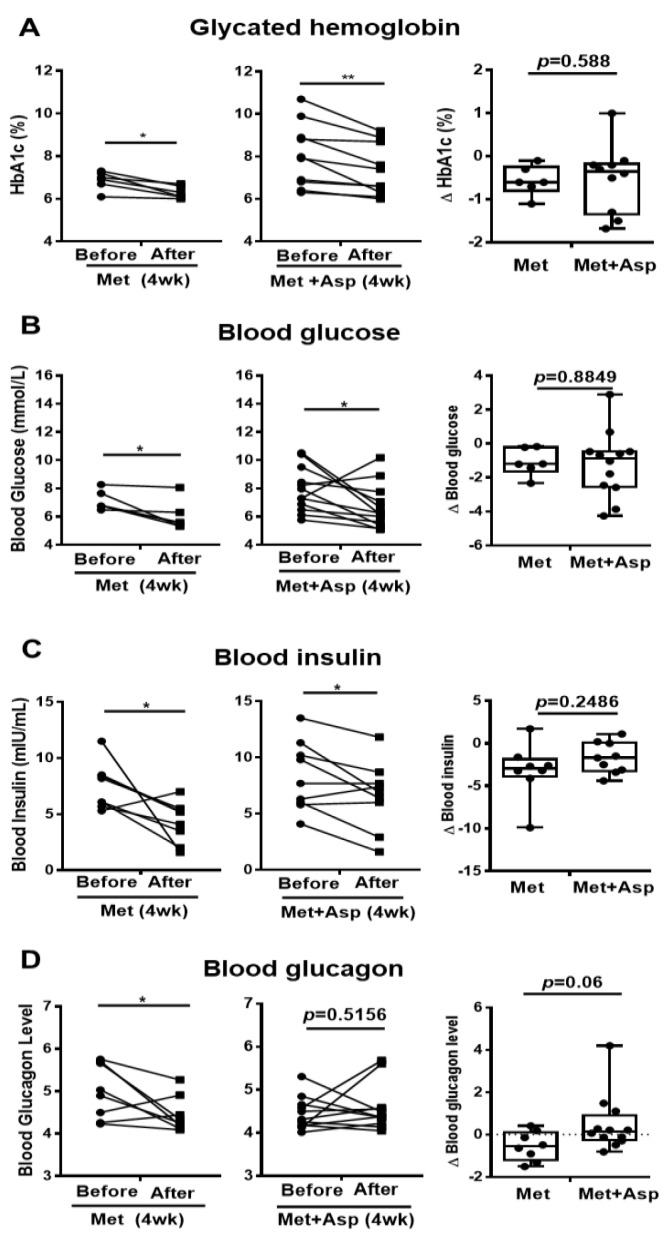
Blood profiles of clinical trial patients with metformin or metformin + aspirin for 4 weeks. (**A**–**D**) The HbA1c (**A**), glucose (**B**), insulin (**C**), and glucagon (**D**) profiles in type 2 diabetic patients’ blood before and after 4 week-treatment of metformin or metformin + aspirin, *n* = 6–12 patients/group. * *p* < 0.05, ** *p* < 0.01.

Patients with T2D were next recruited and treated with either metformin or combined metformin and aspirin (abbreviated hereafter as Group M and Group M + A). Both M and M + A groups exhibited improvement in blood glucose profile, which is indicated by the significant decrease in HbA1c by 0.57 ± 0.14 (%) and 0.72 ± 0.19 (%), respectively, and the significant reduction in blood glucose by 1.10 ± 0.33 (mmol/L) and 1.22 ± 0.57 (mmol/L), respectively; however, aspirin administration did not significantly enhance the effect of metformin on blood glucose profile (Figure 7A,B). Blood insulin was significantly decreased in M group by 3.21 ± 1.14 (mIU/mL) and in M + A group by 1.70 ± 0.61 (mIU/mL), where no additional effect of salicylate was observed (Figure 7C). Metformin treatment group showed a significant reduction in blood glucagon level, which was not observed in metformin and salicylate co-treatment group (Figure 7D).

The serum lipid profiles were also analyzed. Both M and M + A groups showed no significant changes in blood HDL and free fatty acid (FFA) levels (Supplementary Figure S1A,B). Blood triglyceride was significantly decreased in M group but not in M + A group (Supplementary Figure S1C). Both M and M + A groups exhibited marked reduction in blood LDL levels (M: 0.58 ± 0.31 mmol/L; M + A: 0.25 ± 0.15 mmol/L) and no significant differences were found between the effect of M and M + A on blood LDL reduction (Supplementary Figure S1D). These results suggest that both metformin treatment and metformin-aspirin co-treatment improve blood profiles, but aspirin had no significantly additive effect on the metformin action in blood profiles.

## 4. Discussion

Metformin is a well-established anti-hyperglycemic drug for the treatment of diabetes. Despite its success in clinical application, the molecular mechanisms of metformin action remain incompletely understood. One of the reasons may be attributed to the metformin doses used in studies, since most of studies were performed with supra-pharmacological concentrations of metformin. In this study, we analyzed the dosage effect of metformin on HGP and blood glucose. We found that metformin suppresses HGP and blood glucose through the PKA→Foxo1 signaling pathway. Further investigation showed that Foxo1-S273 mutations attenuate metformin-mediated HGP and blood glucose suppression. In addition, salicylate also inhibits HGP partially through Foxo1, but it has no significant additive effect on metformin-suppressed HGP and blood glucose. Finally, we showed that metformin and salicylate treatments inhibit the PKA→Foxo1 signaling pathway and improve glucose homeostasis in HFD-fed mice. This study provides a new mechanism of metformin action, where metformin inhibits PKA activity, decreases Foxo1-S273 phosphorylation, and suppresses HGP (Supplementary Figure S2).

Metformin is the first-line treatment for type 2 diabetes mellitus worldwide. The maximal total daily use of metformin for diabetes treatment is 2.5 g or 35 mg/kg body weight. Metformin is stable and not metabolized in animals; the majority of metformin is eliminated via the renal system [31]. After absorption into the enterocytes, metformin is directly transported to the liver via the portal vein. Under a therapeutic dose administration, the concentration of metformin in portal vein is around 40–70 μM [3]. In our study, we treated primary hepatocytes with 50, 100, and 200 μM of metformin. We did not observe the significant suppressive effect of metformin on glucagon-induced HGP at 50 μM concentration. Cao et al. reported that low metformin concentrations, such as 60 or 80 μM, significantly decreased glucagon-induced HGP [12]. This discrepancy may be explained by the different pretreatment time of metformin. In Cao et al.’s study, primary hepatocytes were pretreated with low dose metformin for 24 h, whereas we only pretreated the primary hepatocytes with metformin for 30 min in this study. Indeed, we found 100 μM of metformin significantly attenuates glucagon-induced HGP; this effect depends on Foxo1 in hepatocytes. High-concentration metformin (>200 μM) decreases ATP levels and increases AMP levels, which directly inhibits hepatic gluconeogenesis [11]. Moreover, protein translation might also be affected under high-concentration metformin due to its non-specific effect [3]. In our study, 200 μM of metformin further decreased glucagon-induced HGP, which is partially blocked by hepatic Foxo1 deficiency. However, 500 μM of metformin largely abolished glucagon-induced HGP in both control and FKO hepatocytes (data not shown), which indicates that high-concentration metformin impairs hepatic energy state or other non-specific targets and bypasses Foxo1 to suppress hepatic gluconeogenesis. Consistently, our mice data indicate that metformin-mediated blood glucose and HGP suppression relies on hepatic Foxo1. Thus, Foxo1 is one of the important targets of metformin to regulate glucose homeostasis under therapeutic concentrations.

We previously reported that glucagon stimulates Foxo1-S273 phosphorylation through PKA activation, increasing Foxo1 activity and promoting HGP [23]. In this study, we found that metformin significantly inhibits glucagon-induced PKA activity in hepatocytes and the inhibition of PKA by H89 blocks the suppression of glucagon-induced HGP by metformin, which indicates that PKA-Foxo1-S273 pathway is a potential target of metformin in regulation of glucagon action. Indeed, Foxo1-S273 mutations blocked metformin-induced HGP and blood glucose suppression in both hepatocytes and mice. More importantly, metformin attenuated the PKA→Foxo1 signaling pathway in HFD-fed mice, which partially accounts for the beneficial effects of metformin in glucose homeostasis. Consistent with previous studies [22,32], glucose tolerance is significantly improved in FKO mice under physiological conditions. The beneficial effect of metformin on glucose tolerance is diminished in FKO mice, further suggesting that Foxo1 plays a key role in metformin-improved glucose homeostasis. The improvement of glucose tolerance in FKO mice is partially attributed to the reduced glucose production. In addition, we previously showed that hepatic mitochondrial function is stimulated [33] and hepatic glucokinase expression level is increased [22] in FKO mice, which promotes glucose oxidation in livers. The increased glucose utilization may contribute to improving glucose tolerance. Moreover, Foxo1 stimulates the secretion of hepatokines, such as follistatin, which targets adipocytes to impair glucose uptakes [34]. Thus, the reduction of hepatokines targeted by Foxo1 may also mediate the improvement in glucose tolerance in FKO mice. The inhibition of PKA activity by metformin may be attributed to the reduced cellular cAMP levels. The low concentration of metformin activates AMPK, which in turn antagonizes glucagon-stimulated cellular cAMP-PKA signaling through PDE4B [9,12]. A high level of metformin, in addition to activating AMPK, promotes the accumulation of AMP, inhibiting adenylate cyclase and reducing cellular cyclic AMP (cAMP) levels and inactivating the PKA–Foxo1 signaling pathway [13]. Foxo1, an important transcription factor for gluconeogenesis, is phosphorylated by many kinases, such as PKA, Akt, p38, and ERK [22,35,36]. Although we identified that Foxo1-S273 phosphorylation is a major target of metformin in regulation of HGP, we cannot exclude the potential roles of other phosphorylation sites in Foxo1, including S284, S295, S326, S467, S475, T24, S246, S253, S413, S415, and T553. Especially, Ozcan et al. found that nine alanine mutations in Foxo1 at S284, S295, S326, S467, S475, S246, S253, S413, and S415 sites impairs Foxo1 nuclear translocation [35]. Further work is needed to evaluate the role of these serine sites’ phosphorylation in the metformin action on glucose regulation.

High doses of salicylate have been used to treat inflammatory conditions mainly through inhibition of nuclear factor kappa B (NF-KB) and IKB kinase β (IKKβ) [36]. High doses of salicylate also lower blood glucose and improve glucose homeostasis [28,29,37]. The beneficial effects of salicylate on glucose control is partially mediated through AMPK activation [17]. Here, we provide evidence that salicylate shares similar target with metformin to suppress hepatic gluconeogenesis through the inhibition of the PKA→Foxo1 signaling pathway. However, we did not observe a significant improvement by salicylate in metformin action on glucose homeostasis in both HFD-fed mice and patients with T2D. In this regard, previous work reported that metformin and salicylate synergistically activate AMPK and improve glucose homeostasis [38]. Considering the metformin concentration matters, the discrepancies between our study and others may be due to the different metformin concentrations used in mice study. We delivered the metformin to mice through drinking water with 0.4 mg/mL metformin, which equals around 1.6 mg/mice/day; whereas in the study of Ford et al., the mice were fed with HFD with 2.5 g/kg metformin, which equals around 7.5 mg metformin/mice/day. This fivefold difference in metformin concentration potentially led to different results in mice. Therefore, it is worth evaluating the effect of the metformin and salicylate combination at different concentrations on glucose homeostasis. Of note, we did observe the beneficial effect of salicylate on blood glucose control in HFD-fed mice, which is partially mediated through the suppression of the PKA→Foxo1 signaling pathway. Although the number of patients is limited in our study, we observed the significant improvement in blood glucose profile and LDL profile in both metformin and metformin + aspirin groups. However, we did not find a better effect of metformin + aspirin on blood glucose profiles, as compared to that in metformin treatment. Interestingly, metformin + aspirin treatment tends to further improve HbA1c and blood glucose compared to the metformin treatment group; however, there is no significant difference due to the small sample size. Therefore, further work is needed to detect the effect of metformin + aspirin treatment on blood glucose profiles in a clinical trial with a large number of patients.

## 5. Conclusions

In this study, we reveal a new mechanism by which metformin suppresses HGP and blood glucose. Metformin inhibits PKA activity, decreases Foxo1-S273 phosphorylation, and attenuates Foxo1 activity, and thus suppresses HGP. In addition, salicylate also reduces HGP through the PKA→Foxo1 signaling pathway, whereas salicylate does not significantly improve the metformin action on glucose homeostasis in both HFD-fed mice and T2D patients.

## Supplementary Materials

The following are available online at https://www.mdpi.com/article/10.3390/biom11060873/s1, Figure S1: Blood lipid profiles of clinical trial patients with metformin or metformin + aspirin for 4 weeks; Figure S2: Mode of metformin and salicylate in regulation of glucose homeostasis in hepatocytes.
