# Supplementary material for: Metformin Targets Foxo1 to Control Glucose Homeostasis
Source: Biomolecules. 2021 Jun 11;11(6):873. doi: 10.3390/biom11060873 (PMC8231152; doi:10.3390/biom11060873)
Supplement: Supplementary file 1 [file biomolecules-11-00873-s001.zip › biomolecules-1236418-supplementary.pdf]

Supplementary Figure S1

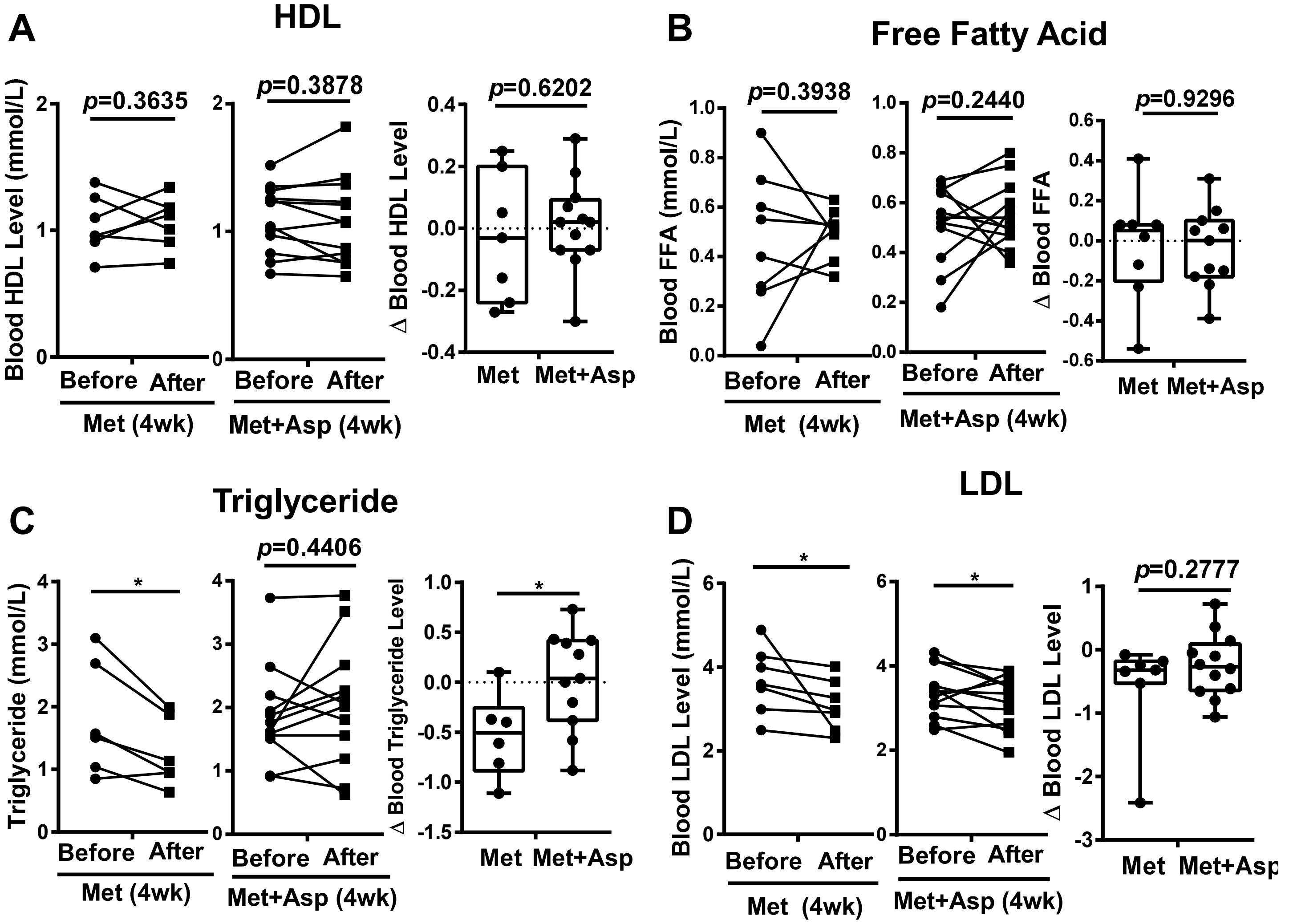

Supplementary Figure S1. Blood lipid profiles of clinical trial patients with metformin or metformin + aspirin for 4 weeks. **(A-D)** The HDL (A), free fatty acid (B), triglyceride (C) and LDL (D) profiles in type 2 diabetic patients' blood before and after 4 week-treatment of metformin or metformin + aspirin, n=6-12 patients/group. * $p < 0.05$.

Supplementary Figure S2

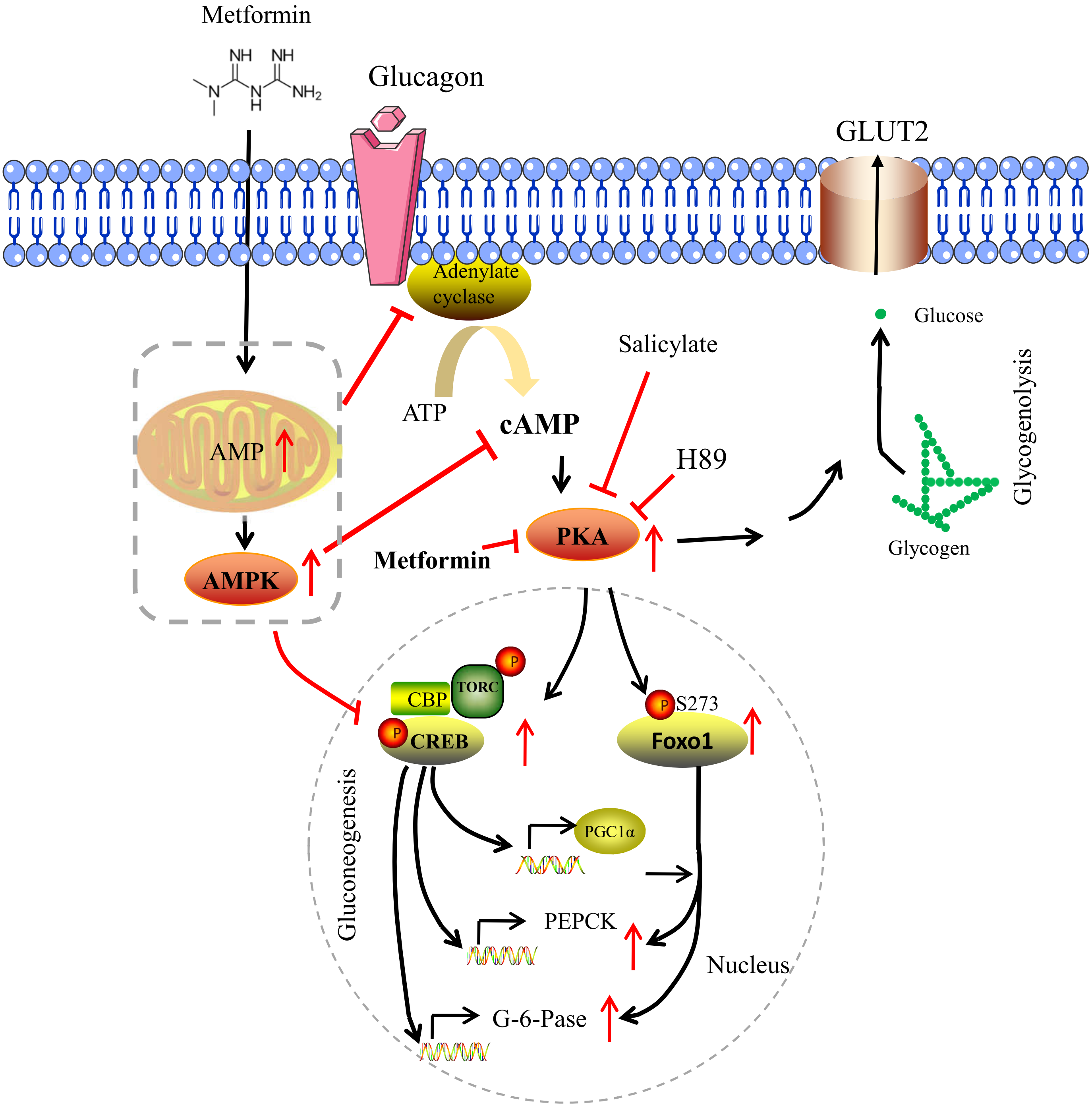

Supplementary Figure S2. Mode of metformin and salicylate in regulation of glucose homeostasis in hepatocytes. Metformin inhibits PKA activity, decreases Foxo1-S273 phosphorylation, and suppresses hepatic gluconeogenesis. Salicylate also reduces HGP through the inhibition of PKA→Foxo1-S273 signaling pathway.
